# Effect of platelet-rich plasma on the degenerative rotator cuff tendinopathy according to the compositions

**DOI:** 10.1186/s13018-019-1406-4

**Published:** 2019-12-02

**Authors:** Sang Jun Kim, Seung Mi Yeo, Soo Jin Noh, Chul-Won Ha, Byung Chan Lee, Hyo Sun Lee, Sun Jeong Kim

**Affiliations:** 1 Seoul Jun Rehabilitation Research Center, Seoul Jun Rehabilitation Medical Center, Nambusoonhwanro, 2606, Seoul, 06737 South Korea; 2 Department of Physical and Rehabilitation Medicine, Stem Cell & Regenerative Medicine Institute, Samsung Medical Center, Sungkyunkwan University School of Medicine, Seoul, 06351 South Korea; 3 Department of Orthopaedic Surgery, Stem Cell & Regenerative Medicine Institute, Samsung Medical Center, Sungkyunkwan University School of Medicine, Seoul, 06351 South Korea

**Keywords:** Platelet-rich plasma, Rotator cuff, Exercise, Growth factors, Shoulder function

## Abstract

**Background:**

There are controversies about platelet-rich plasma (PRP) as an established treatment option for rotator cuff (RC) tendinopathy. The purpose of the study was to find the relation of cellular component with clinical efficacy in RC tendinopathy and to find the composition of PRP in treating RC tendinopathy.

**Methods:**

A total 30 patients were recruited and divided into PRP and control groups. In the PRP group, 2 ml of PRP solution was injected to the hypoechoic lesion of degenerative supraspinatus via 22-gauge syringe with peppering technique. Patients in the control group were taught rotator cuff strengthening exercises. American Shoulder and Elbow Surgeons (ASES), Constant-Murley score, and numeric rating scale (NRS) were measured before, 6 weeks after, 12 weeks after, and 24 weeks after the procedure. PRP compositions were analyzed using the 1 ml of PRP solution.

**Results:**

Linear regression analysis showed no significant difference of ASES and Constant-Murley scores between the groups at 6 weeks (*P* = 0.582 and 0.258) and at 12 weeks (*P* = 0.969 and 0.795) but showed a significant difference at 24 weeks (*P* = 0.050 and 0.048). Independent *t* test showed significant group difference of NRS at 6 weeks (*P* = 0.031) but not at 12 and 24 weeks (*P* = 0.147 and 0.935). 5.19 pg/ml in IL-1β and 61.79 μg/ml in TGF-β1 were acquired as cutoff values to predict meaningful improvement. The PRP subgroup above IL-1β or TGF-β1 cutoff value showed significant differences in all clinical outcomes compared with the exercise group while the PRP subgroup below the cutoff value showed no significant differences in linear regression analysis.

**Conclusions:**

Our study can help to find the optimal PRP condition and to enhance the effect of PRP on RC tendinopathy.

**Trial registration:**

All the patients were registered in our Institutional Ethics Committee (approval number 2014-05-009).

## Introduction

Rotator cuff (RC) tendinopathy is a degenerative musculoskeletal disease caused by overuse. The prevalence of RC disease, specifically partial and full thickness RC tendon tears, increases as a function of age starting at 40 years [1] and may be high as 50% by the age of 70 years [2].

Conservative treatments for RC tendinopathy include exercise, medication, and injection therapy. Medication and steroid injection therapy only help to reduce the pain, but exercise is effective as a treatment for regaining function in addition to reduction of pain in RC tendinopathy [3]. However, exercise takes a long time for recovery, and patient compliance is needed to attain maximal effect [4]. Other conservative therapies, such as prolotherapy and ESWT, have been tried to regain function for RC tendinopathy but the clinical evidence is not yet clear [5, 6]. There remains no consensus in conservative therapy except exercise.

Recently, autologous growth factors like platelet-derived growth factor and vascular endothelial growth factor found in platelet-rich plasma (PRP) have been established to play a critical role in cell proliferation, chemotaxis, cell differentiation, and angiogenesis [7]. Several studies have reported favorable clinical outcomes with the use of PRP in the treatment of acute and chronic tendinopathies [8–12] and rotator cuff tears [13]. In contrast to the positive potential of PRP reported in the basic research literature, clinical outcomes have been reported as better [14–16], not different [17–20], or even worse [21] in RC tendinopathy. Therefore, there are controversies about PRP as an established treatment option for RC tendinopathy [22].

In our thought, these studies used different PRP producing methods, which resulted in differences in platelet concentrate and other compositions [23] and diverse clinical outcomes. In addition, these differences in cellular composition can affect regeneration effect benefits. A recent study documented that leukocytes in PRP increased catabolic signaling molecules such as matrix metalloproteinase-9 (MMP-9) and interleukin-1β (IL-1β) [24] and these catabolic proteases can perpetuate inflammation and inhibit tissue healing [25]. Dohan commented that PRP was classified as four main families based on their fibrin architecture and cell content, which affects clinical effects [26]. Therefore, we think that it is important to understand the composition of PRP to enhance tendon healing in RC tendinopathy.

PRP treatment has many advantages in terms of relative safety, easy production, and cost-effectiveness. The purpose of the study was to find the relation of cellular component with clinical efficacy in RC tendinopathy and to find the composition of PRP in treating RC tendinopathy.

## Material and methods

### Study design

This was a prospective, open-label comparative study, designed to ascertain local application of autologous PRP to RC tendinopathy. Our Institutional Ethics Committee approved the study (2014-05-009) and all patients gave written informed consent to participate in this clinical study.

### Patient enrollment

Patients were recruited from the outpatient clinic for shoulder pain and were selected when showing a focal hypoechoic or anechoic defect in the tendons on ultrasound (US) examination to diagnose RC tendinopathy. A linear probe (model no. 11 L-D, 3.0–12.0 MHz) in a US machine (Voluson E6®, Siemens, Munchen, Germany) was used for this examination.

Inclusion criteria were (1) persons aged 18 years and older; (2) RC tendinopathy confirmed by ultrasonographic finding; (3) patient agreement to stop medication associated with RC tendinopathy such as NSAIDs except the drugs given by the study for rescue medication; (4) had not undergone shoulder surgery within 3 months; (5) had not taken local steroid injection within 1 month or systematic steroid therapy within 2 weeks; (6) had neither infectious disease nor acute inflammatory disease such as local infection at the site of the procedure; and (7) gave written informed consent. RC tendinopathy was diagnosed when there was a focal hypoechoic or anechoic defect in the tendon of rotator cuff muscles [27].

Exclusion criteria were (1) persons younger than 18 years; (2) had a platelet dysfunction syndrome or platelet count lower than 10^5^/μl which is inappropriate for extraction or injection of PRP; (3) had active septicemia or local infection at the site of the procedure; (4) had taken local steroid injection within 1 month or systematic steroid therapy within 2 weeks; (5) had fever or infectious disease within 2 weeks; (6) had history of cancer or history of cancer therapy such as chemotherapy within 1 year; (7) currently pregnant or lactating women; or (8) could not voluntarily write subject record due to cognitive dysfunction.

Patients were divided into two groups, the PRP group and the exercise group, in a consecutive order. The PRP group received PRP injection one time while the exercise group followed self-exercise programs for strengthening rotator cuff tendon.

### Autologous platelet-rich plasma (PRP) preparation

**Fig. 1:**
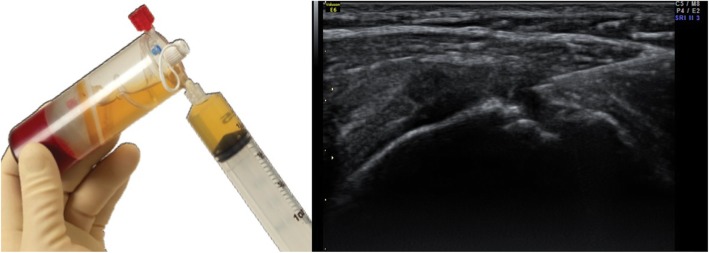
A total of 2 ml of PRP solution was injected to the hypoechoic lesion of degenerative supraspinatus via 22-gauge syringe with peppering technique

Twenty milliliters of whole blood was drawn from patients with an 18-gauge syringe filled with citrate anticoagulant (ACD-A) using a sterile technique. The anticoagulated blood was then transferred to a specially designed disposable tube, which was placed in a centrifuge (GPS® III – Plasmax- Platelet Concentration System; Biomet Biologics, Warsaw, IN, USA) for 15 min at 3200 rpm. The concentrated platelets, on top of the floating buoy, were stored in a sterile syringe (Fig. 1).

### Injection of PRP

Patients were in supine position with their arms placed on the superior aspect of the iliac wing with the palm up and the elbow flexed. We found the long head of biceps in the intertubercular groove transversely via ultrasound. After lining the probe along the long axis of biceps tendon, the probe was moved to the supraspinatus tendon in a parallel position. After finding the hypoechoic lesion, 2 ml of PRP solution was injected to the hypoechoic lesion of degenerative supraspinatus via 22-gauge syringe with peppering technique (Fig. 1). Peppering technique was used to avoid tendon morphology disruption by injecting PRP into the tendon [28]. The remaining 1 ml of PRP solution was used in analyzing the compositions of PRP.

### Rotator cuff strengthening exercise

Patients in the control group were taught rotator cuff strengthening exercises by one experienced physical therapist. The rotator cuff strengthening exercises consisted of scapular stabilizing exercise and infraspinatus and subscapularis strengthening isometric and isotonic exercise using dumbbell and Thera-Band. They also received a brochure containing rotator cuff strengthening protocol (a Additional file 1: Figure S1) and were asked to perform this for 20 min at least 4 days per week by themselves.

Exercise compliance was measured at each visit by asking how many times the patient followed exercise and was calculated by days following exercise per week divided by four and multiplied by 100 (%).

Patients in the PRP group received the same brochure but were not asked to follow the exercise and exercise compliance was not measured.

### Evaluation

Each patient was evaluated before, 6 weeks after, 12 weeks after, and 24 weeks after the procedure. American Shoulder and Elbow Surgeons (ASES), Constant-Murley score, and numeric rating scale (NRS) were used as outcome measures. Thickness of the rotator cuff tendon measured by US was estimated before and 24 weeks after the procedure.

ASES was used in a version previously described [29]. The Constant-Murley score was calculated following a detailed physical examination in a standardized fashion. Active strength in forward flexion for the Constant-Murley score was tested as an average of 3 pulls in 90° of abduction in the scapular plane. The wrist was fixed in pronation, with the hand facing the floor and elbow fully extended. Subjects were instructed to pull upward with maximal effort until requested to stop. Patients with active abduction of < 90° were given 0 points for strength.

In addition to these measures, all local or general complications during the procedure or follow-up phases were recorded.

### Ultrasound assessment

Rotator cuff tendon integrity was evaluated by US imaging examination before and 24 weeks after the procedure. US examinations were performed with the patient seated on a backless chair. The patient was positioned with his/her arm placed posteriorly, placing the palmar side on the superior aspect of the iliac wing with the elbow flexed posteriorly. By positioning the transducer around the curvature of the humeral head in the transverse plane, the biceps was viewed in the osseous groove. Once located, the transducer was rotated until the biceps was viewed longitudinally. The transducer was moved in a parallel direction to show the long axis of the supraspinatus tendon. Thickness of the supraspinatus was measured at the greatest distance (mm) among the long axis images of the supraspinatus tendon. 

### Growth factor composition analysis

In the PRP group, TGF-β1, and TNF-α, PDGF-AA, PDGF-AB, PDGF-BB, VEGF, EGF, IGFBP-1, IL-1β, and IL-8 were analyzed using the 1 ml of PRP solution. The analysis was performed by Mag LxPA Base Kit (catalog no. LTGM00, 100,200,300, Koma Biotech, Seoul, Korea). After PRP solution centrifugation, the supernatant was diluted at 1/15. Antibodies of growth factors were added and mixed at the room temperature for 2 h, and the response was measured by Luminex (Luminex, Austin, TX, USA). The median fluorescent intensity was selected after acquiring the standard curves according to the concentration by MasterPlex QT2010 (MiraiBio, Hitachi, CA, USA) using the best fit method. These experiments were duplicated, and the average value was used for the concentration of growth factors.

### Sample size estimation

Sample size was calculated based on a previous study that demonstrated the effect of corticosteroid injection on rotator cuff tendinopathy [30]. Average difference of Constant-Murley scores between the groups was 17.7 and the estimated standard deviation was 21.4. Considering an α-error of 0.05 and a β-error of 0.1, the minimum sample size needed was 26.4. Therefore, the necessary sample size in each group was 30 considering that the dropout rate was 10%.

### Statistics

Changes of the ASES, Constant-Murley score, and NRS were compared at each time interval by paired *t* test to show significant change before and after the intervention and by independent *t* test to show significant differences between groups.

Correlations of changes in clinical outcomes with PRP compositions in the PRP group were analyzed by the Pearson correlation coefficient. After selection of PRP compositions that showed significant correlation with clinical outcomes, receiver operating characteristic (ROC) curve was drawn to acquire the cutoff value of PRP compositions to predict meaningful improvement in clinical outcomes. We set the meaningful improvement in ASES as increase greater than 27 based on a previous study [31], in Constant-Murley score as an increase greater than 18 based on a previous study [32], and in NRS as a decrease greater than four based on a previous study [33].

Patients in the PRP group were divided into two groups according to the cutoff value and these subgroups were again compared with the exercise group by independent *t* test.

The main statistical analysis was based on intention-to-treat analysis with worst-case imputation for lost data and was supported by additional per protocol analysis. Additional per protocol analysis was repeated to enhance the results. SPSS 23.0 software (SPSS Inc, Chicago, IL, USA) was used for statistical analysis.

## Results

A total of 30 patients were recruited from April 2015 to January 2017. Table 1 shows the initial baseline characteristics of the patients. In the PRP group, three patients were lost to follow-up from 6 weeks after the PRP injection, one patient was lost to follow-up from 12 weeks after the procedure, and four patients were lost to follow-up from 24 weeks after the procedure. In the exercise group, five patients were lost to follow-up from 6 weeks after the PRP injection, two patients were lost to follow-up from 12 weeks after the procedure, and no patients were lost to follow-up from 24 weeks after the procedure. Lost data was managed by the worst-case imputation method. Among patients lost to follow-up in the PRP group, one patient paradoxically increased their pain after PRP injection and was diagnosed as having newly developed adhesive capsulitis. Exercise compliance in the exercise group was 90.8 ± 30.6% at 6 weeks, 94.2 ± 37.4% at 12 weeks, and 87.4 ± 39.0% at 24 weeks.

**Table 1:** Baseline characteristics of patients in PRP and exercise groups

	PRP group	Exercise group	*P* value
Age (years)	55.6 ± 5.5	53.7 ± 11.5	0.682
Sex (men/women)	14/16	19/11	0.299
Height (cm)	164.7 ± 8.2	167.2 ± 9.4	0.260
Body weight (kg)	66.1 ± 10.9	66.2 ± 10.1	0.807
ASES	42.8 ± 18.4	59.0 ± 13.4	< 0.001*
Constant-Murley score	66.5 ± 17.7	80.9 ± 11.6	0.004*
NRS	5.7 ± 2.3	4.8 ± 1.6	0.110
Tendon thickness (mm)	8.6 ± 9.4	5.6 ± 0.9	0.086

*P value less than 0.05

**Fig. 2:**
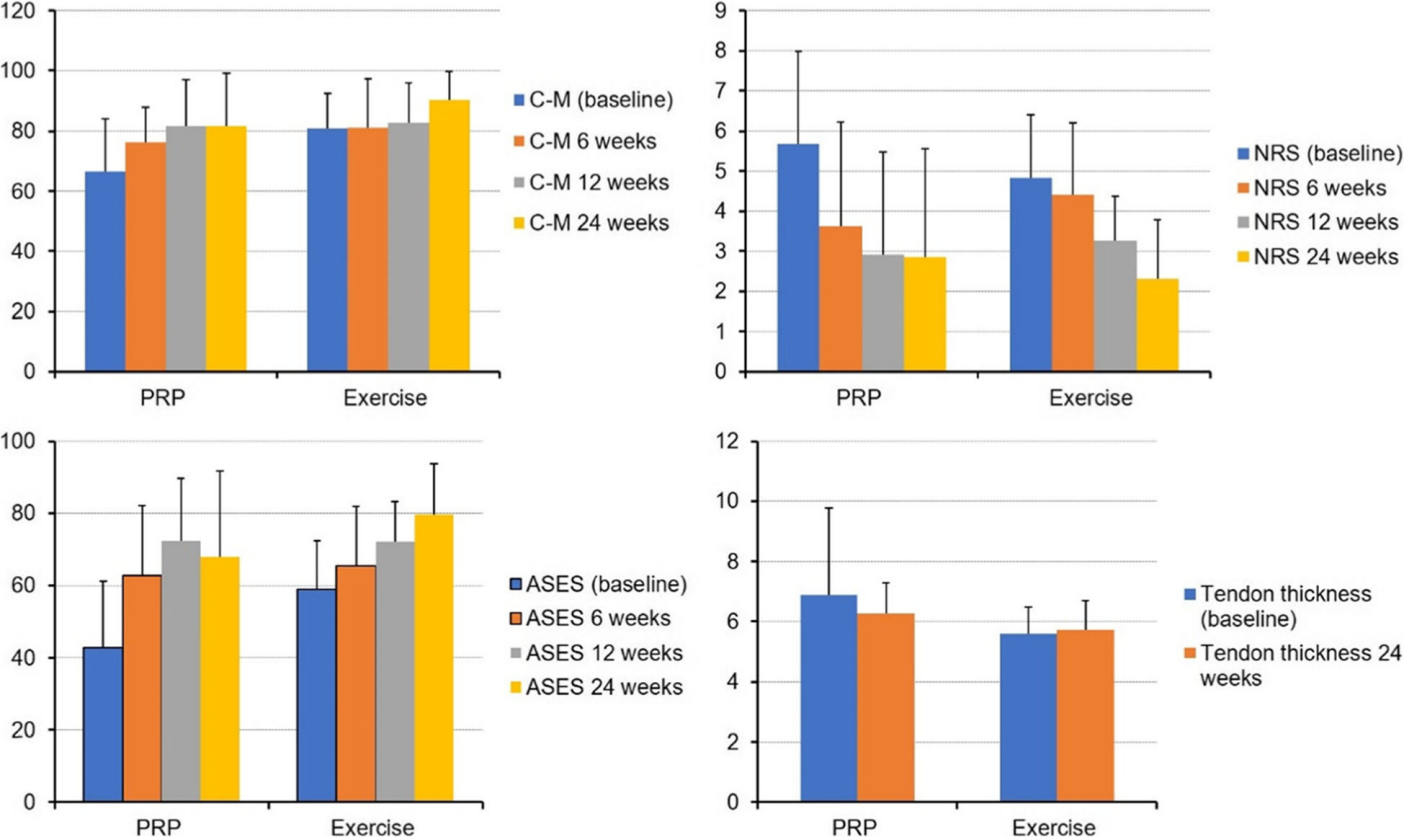
Linear regression analysis showed no significant difference of ASES scores between the groups at 6 weeks (*P* = 0.582) and at 12 weeks (*P* = 0.969) but showed a significant difference at 24 weeks (*P* = 0.050)

ASES scores in the PRP group changed from 42.8 ± 18.4 to 62.7 ± 19.4 at 6 weeks (*P* < 0.001, paired *t* test), 72.4 ± 17.3 at 12 weeks (*P* < 0.001, paired *t* test), and 68.0 ± 23.8 at 24 weeks after PRP injection (*P* = 0.003, paired *t* test). ASES scores in the exercise group changed from 59.0 ± 13.4 to 65.4 ± 16.4 at 6 weeks (*P* < 0.012, paired *t* test), 72.3 ± 11.0 at 12 weeks (*P* < 0.001, paired *t* test), and 79.7 ± 14.1 at 24 weeks after exercise (*P* < 0.001, paired *t* test). Because baseline ASES scores showed significant difference between the PRP and exercise groups, linear regression analysis was conducted for adjustment of baseline ASES scores. Linear regression analysis showed no significant difference of ASES scores between the groups at 6 weeks (*P* = 0.582) and at 12 weeks (*P* = 0.969) but showed a significant difference at 24 weeks (*P* = 0.050; Fig. 2). Per protocol analysis showed the same results with intention-to-treat analysis with worst-case imputation.

Constant-Murley scores in the PRP group changed from 66.5 ± 17.7 to 76.3 ± 14.9 at 6 weeks (*P* = 0.008, paired *t* test), 81.6 ± 15.3 at 12 weeks (*P* = 0.001, paired *t* test), and 81.7 ± 17.4 at 24 weeks after PRP injection (*P* = 0.022, paired *t* test). Constant-Murley scores in the exercise group changed from 80.9 ± 11.6 to 81.2 ± 16.1 at 6 weeks (*P* = 0.787, paired *t* test), 82.7 ± 13.3 at 12 weeks (*P* = 0.953, paired *t* test), and 90.2 ± 9.5 at 24 weeks after exercise (*P* = 0.003, paired *t* test). Because baseline Constant-Murley scores showed significant difference between the PRP and exercise groups, linear regression analysis was conducted for adjustment of baseline Constant-Murley scores. Linear regression analysis showed no significant difference of Constant-Murley scores between groups at 6 weeks (*P* = 0.258) and at 12 weeks (*P* = 0.795) but showed a significant difference at 24 weeks (*P* = 0.048; Fig. 2). Per protocol analysis showed the same results with intention-to-treat analysis with worst-case imputation.

NRS scores in the PRP group changed from 5.7 ± 2.3 to 3.6 ± 2.6 at 6 weeks (*P* = 0.004, paired *t* test), 2.9 ± 2.6 at 12 weeks (*P* = 0.003, paired *t* test), and 2.9 ± 2.7 at 24 weeks (*P* = 0.007, paired *t* test) after PRP injection. NRS scores in the exercise group changed from 4.8 ± 1.6 to 4.4 ± 1.8 at 6 weeks (*P* = 0.202, paired *t* test), 3.3 ± 1.1 at 12 weeks (*P* < 0.001, paired *t* test), and 2.3 ± 1.5 at 24 weeks (*P* < 0.001, paired *t* test) after exercise. Independent *t* test showed significant group difference at 6 weeks (*P* = 0.031) but not at 12 and 24 weeks (*P* = 0.147 and 0.935). Per protocol analysis showed the same results with intention-to-treat analysis with worst-case imputation.

Thickness of the supraspinatus in the PRP group decreased 6 months after PRP injection while thickness in exercise group slightly increased 24 weeks after exercise.

**Fig. 3:**
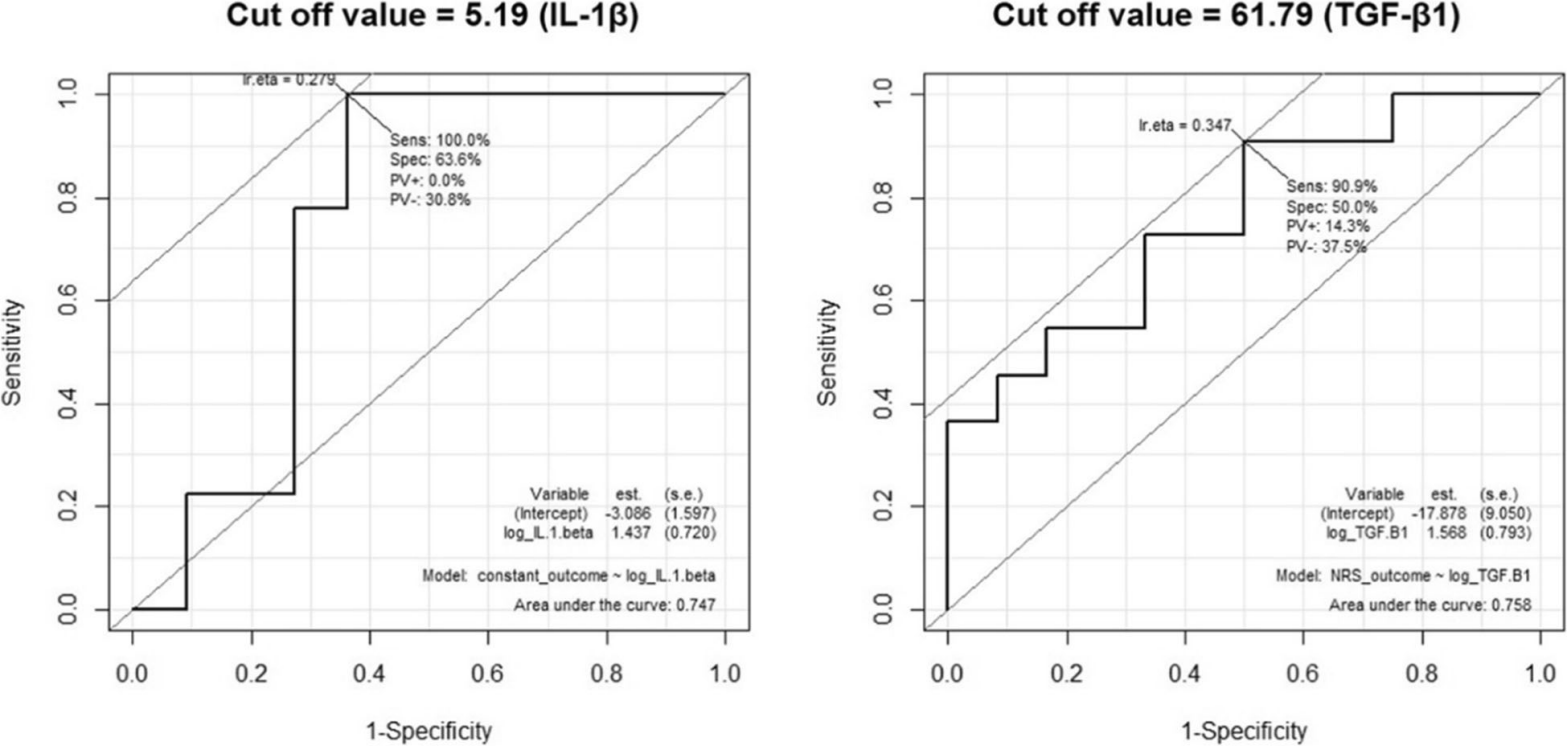
ROC curves were drawn to acquire cutoff values of IL-1β and TGF-β1 using meaningful improvement of Constant-Murley score and NRS

The distribution of PRP components is presented in Additional file 2: Figure S2. The correlation of PRP components with changes of ASES, Constant-Murley score, and NRS showed that significant correlation was found between IL-1β and change of Constant-Murley score at 12 weeks (*P* = 0.046), and between TGF-β1 and change of NRS at 12 weeks (*P* = 0.048; Table 2). ROC curves were drawn to acquire cutoff values of IL-1β and TGF-β1 using meaningful improvement of Constant-Murley score and NRS (Fig. 3) and 5.19 pg/ml in IL-1β and 61.79 μg/ml in TGF-β1 were acquired as cutoff values to predict meaningful improvement.

**Table 2:** Correlation between PRP compositions and clinical outcomes

	NRS		Constant-Murley		ASES	
	*z* value	Pr(>|*z*|)	*z* value	Pr(>|*z*|)	*z* value	Pr(>|*z*|)
log_CXCL8/IL-8	0.663	0.508	0.628	0.530	0.138	0.891
log_EGF	1.196	0.232	1.165	0.244	− 0.174	0.862
log_IGFBP-1	1.653	0.098	0.067	0.947	0.720	0.472
log_IL-1β	1.816	0.069	1.995	0.046*	− 0.319	0.750
MMP-13	0.096	0.924	− 0.799	0.424	− 0.693	0.488
log_PDGF-AB	0.257	0.797	− 0.161	0.872	1.293	0.196
log_TNF-α	1.377	0.169	0.977	0.328	0.086	0.932
log_VEGF	1.455	0.146	1.450	0.147	− 0.118	0.906
log_PDGF-AA	1.250	0.211	1.186	0.236	0.009	0.993
log_PDGF-BB	0.795	0.427	0.758	0.448	− 0.252	0.801
log_TGF-β1	1.977	0.048*	1.246	0.213	1.169	0.242
log_TGF-β2	1.334	0.183	0.558	0.577	0.851	0.395

*P value less than 0.05

Patients in the PRP group were divided into two subgroups according to the cutoff values for IL-1β and TGF-β1 and clinical outcomes in these two subgroups were again compared with those in the exercise group. The PRP subgroup above the TGF-β1 cutoff value showed significant difference in ASES and Constant-Murley score compared with the exercise group while the PRP subgroup below cutoff value showed no significant difference in linear regression analysis (Table 3). The PRP subgroup above IL-1β cutoff value showed significant differences in all clinical outcomes compared with the exercise group while the PRP subgroup below the cutoff value showed no significant differences in linear regression analysis (Table 3).

**Table 3:** Comparison of clinical outcomes at three months between PRP subgroups and exercise group

	*t* value	Pr(>|*t*|)
TGF-β < 61.79 μg/ml		
ASES	− 0.173	0.864
Constant-Murley score	− 0.474	0.639
NRS	− 0.274	0.786
TGF-β > 61.79 μg/ml		
ASES	− 2.465	0.018*
Constant-Murley score	− 2.350	0.024*
NRS	1.871	0.069
IL-1β < 5.19 pg/ml		
ASES	− 0.475	0.638
Constant-Murley score	− 0.195	0.847
NRS	− 0.105	0.917
IL-1β > 5.19 pg/ml		
ASES	− 2.664	0.012*
Constant-Murley score	− 3.345	0.002*
NRS	2.370	0.023*

* asterisk means *p*-value less than 0.05

## Discussion

In this study, we found that TGF-β1 and IL-1β among cellular components of PRP were related to clinical efficacy for RC tendinopathy and concentration of IL-1β above 5.19 pg/ml and TGF-β1 above 61.79 μg/ml in PRP had better clinical outcomes for RC tendinopathy than the exercise group.

ASES score improved about from 10 to 20 in Cai’s study [34], from 10 to 30 in Kim’s study [35], and from 20 to 30 in Shams’ study [36] after PRP injection for partial tear of the rotator cuff tendon, which are similar to the results in our study. Constant-Murley score improved about 40 in Holtby’s study [37], from 15 to 25 in Shams’ and von Wehren’s studies [36, 38], which are similar to the results in our study. Although significant improvement in clinical outcomes after PRP injection was found to be similar to prior studies, this improvement was not superior to exercise. Considering that exercise must be continuously followed for several months to acquire desired clinical outcomes, PRP injection has an advantage over exercise in terms of 109pt?>In terms of time course, all clinical outcomes in the PRP group showed gradual improvement until 12 weeks but plateau or slight decrease at 24 weeks while those in the exercise group showed gradual improvement until 24 weeks and the greatest improvement was found at 24 weeks. This can be explained by the fact that PRP injection was done just one time without additional following injections, so the effect of PRP was diminished while exercise was maintained until 24 weeks, so the exercise effect was cumulative. We think that repeated injection of PRP is necessary to enhance the effect of PRP on RC tendinopathy.

The thickness of the supraspinatus was decreased at 24 weeks after PRP injection but was increased at 24 weeks after exercise. Tendon thickness in degenerative tendinopathy was seen to be increased on ultrasound examination [39] and decreased after adequate treatment [40], which can explain our results. Tendon thickness is known to be increased after exercise [41], which also explains our results.

IL-1β and TGF-β1 were correlated with clinical outcomes while other PRP components were not. IL-1β is a major cytokine that induces catabolic action on tendon fibroblasts via the upregulation of inflammatory mediators and plays a role in the tendon’s degenerative changes in tendinopathy [42] or regenerative capacity [43]. These roles might be affected by the concentration of IL-1β and our study showed IL-1β above 5.19 pg/ml had positive effects. TGF-β1 inhibits MMP-9 and MMP-13 expression to increase collagen accumulation [44] and is known to improve tendon strength and tendon healing [45, 46]. Our study demonstrated that TGF-β1 above 61.79 μg/ml was related to functional improvement in RC tendinopathy.

Okamura et al. demonstrated that IL-8 had a positive correlation with shoulder function improvement [47]. In other studies, PDGF, VEGF, and EGF, which are known growth factors in PRP, showed a positive correlation with shoulder function but our study did not show any significant correlation [48, 49]. This might be due to different concentration levels of these growth factors.

Unfortunately, baseline ASES and Constant-Murley scores in the exercise group were higher than those in the PRP group, which might cause biased results. To minimize this bias, baseline adjustment was done through linear regression analysis. However, to validate our results, further studies with larger sample sizes and randomized control designs are needed.

We did not analyze leukocyte concentration in the PRP because our study was focused on growth factors in the PRP. Several studies showed that leukocyte-rich PRP showed harmful effects on tendon healing while leukocyte-poor PRP showed beneficial effects [50–52]. Hilber et al. [50] reported that leukocyte-reduced PRP stimulates the proliferation of tenocytes and Zhang et al. [51] demonstrated that proliferation of tendon stem cells cultured in leukocyte-rich was significantly decreased and tendon stem cells cultured in leukocyte-poor PRP produced more collagen and formed tendon-like tissue. However, these were in vitro studies and clinical studies did not show any significant difference between leukocyte-rich and leukocyte-poor PRPs [53, 54].

Plasma IL-1β and TGF-β levels vary according to the patient condition. Plasma IL-1β level, proinflammatory marker, is higher in rheumatoid arthritis patients [55] than in normal controls and TGF-β, anti-inflammatory marker, also increases in chronic obstructive pulmonary disease patients [56] or in multiple sclerosis [57]. Basic physical condition of patients like these might affect the IL-1β and TGF-β level in the PRP. However, these changes are less than 10-folds, while changes from the concentration of PRP are more than 1000 times. We think that concentration procedure during PRP preparation would affect the IL-1β and TGF-β level in the PRP more strongly than basic physical condition of patients.

In this study, we found IL-1β above 5.19 pg/ml in 58.3% of patients and TGF-β above 61.79 μg/ml in 61.2% of patients. PRP compositions might be affected by the centrifuge protocols [58] and kits used rather than patient characteristics. We used the GPS® III kit and this kit takes PRP from the buffy coat while other kits including ACP® (Device Technologies, MA, USA) take PRP from plasma [23]. In our study, baseline laboratory results including white blood cell and platelet counts from patients were not related to PRP compositions. Smoking and alcohol history did not have any correlation with the PRP compositions.

In our study, exercise compliance was measured by asking how many times the patient followed exercise. Considering that quality of measures of exercise adherence in musculoskeletal diseases is low [59], exercise compliance measures in our study would be limited. Further study to improve measurement of exercise compliance will be necessary.

## Conclusions

We found that TGF-β1 and IL-1β among cellular components of PRP were related to clinical efficacy for RC tendinopathy and concentration of IL-1β above 5.19 pg/ml and TGF-β1 above 61.79 μg/ml in PRP had better clinical outcomes for RC tendinopathy than the exercise group. Despite several limitations, our study can help to find the optimal PRP condition and to enhance the effect of PRP on RC tendinopathy.

## Supplementary information

**Additional file 1:** **Figure S1.** A brochure containing rotator cuff strengthening exercise was delivered to the patients in the control group and they were asked to perform this for 20 minutes at least four days per week by themselves.

**Additional file 2:** **Figure S2.** Platelet rich plasma components including TGF-β1, TNF-α, PDGF-AA, PDGF-BB, PDGF-AB, EGF, IGF-β, VEGF, IL-8, and IL-1β were analyzed and their distribution is presented.

## Data Availability

The datasets used and/or analyzed during the current study are available from the corresponding author on reasonable request.

## Abbreviations

RC: Rotator cuff
PRP: Platelet-rich plasma
MMP-9: Matrix metalloproteinase-9
IL-1β: Interleukin-1β
US: Ultrasound
ASES: American Shoulder and Elbow Surgeons
NRS: Numeric rating scale
ROC: Receiver operating characteristic
