# Supplementary figures and images for: Effect of platelet-rich plasma on the degenerative rotator cuff tendinopathy according to the compositions
Source: J Orthop Surg Res. 2019 Dec 2;14:408. doi: 10.1186/s13018-019-1406-4 (PMC6889570; doi:10.1186/s13018-019-1406-4)

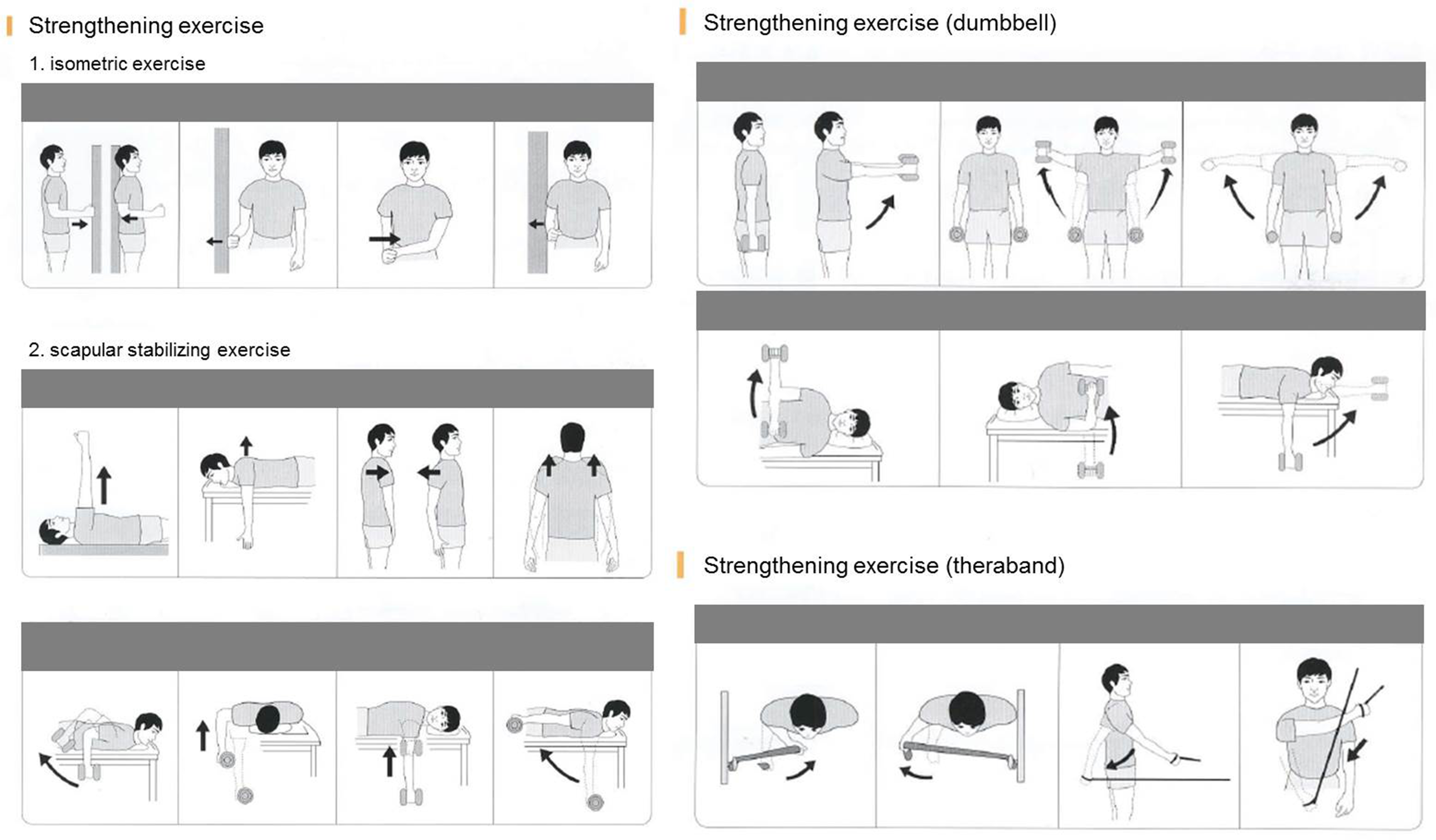

Supplement: Supplementary file 1 — Additional file 1: Figure S1. A brochure containing rotator cuff strengthening exercise was delivered to the patients in the control group and they were asked to perform this for 20 minutes at least four days per week by themselves. [file 13018_2019_1406_MOESM1_ESM.tif]

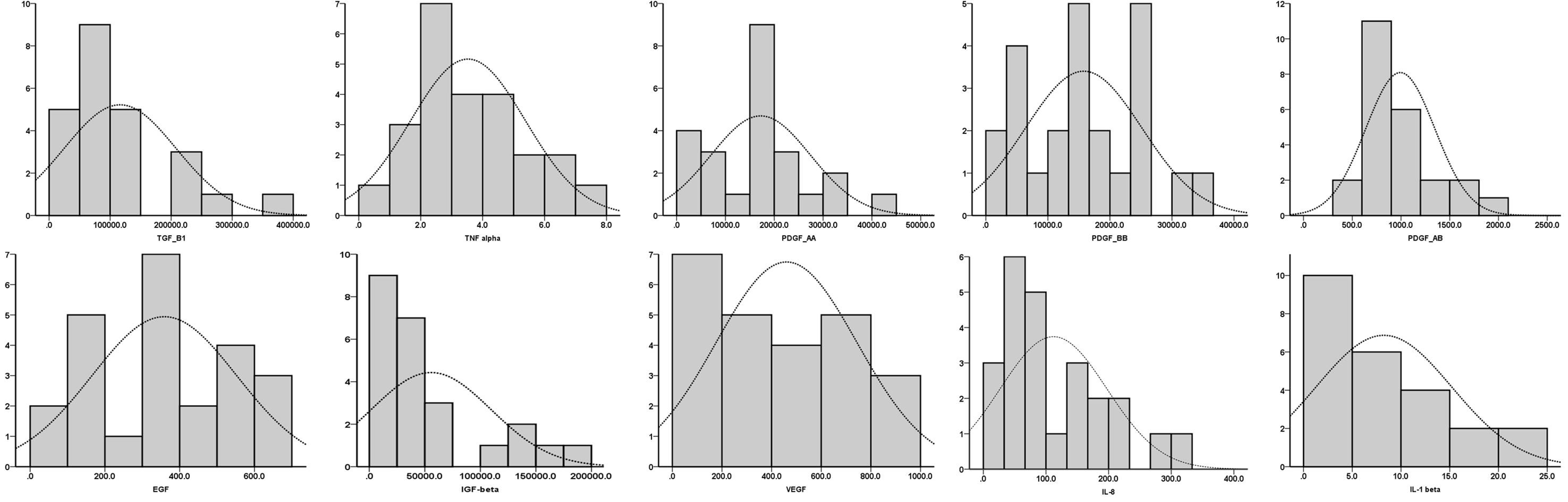

Supplement: Supplementary file 2 — Additional file 2: Figure S2. Platelet rich plasma components including TGF-β1, TNF-α, PDGF-AA, PDGF-BB, PDGF-AB, EGF, IGF-β, VEGF, IL-8, and IL-1β were analyzed and their distribution is presented. [file 13018_2019_1406_MOESM2_ESM.tif]
